# Primary pulmonary meningioma with associated multiple micronodules: a case report and literature review

**DOI:** 10.1093/jscr/rjad034

**Published:** 2023-02-06

**Authors:** Bei Yang, Jingxin Qiu

**Affiliations:** Department of Pathology and Anatomical Sciences, Jacobs School of Medicine and Biomedical Sciences, University at Buffalo, Buffalo, NY, USA; Department of Pathology and Laboratory Medicine, Roswell Park Comprehensive Cancer Center, Buffalo, NY, USA

**Keywords:** primary pulmonary meningioma, FDG PET, multiple micronodules

## Abstract

Primary pulmonary meningioma (PPM) is a rare and benign slow growing tumor with good prognosis. It often presents as an asymptomatic, well-circumscribed, solitary pulmonary nodule. Wedge resection is the management of choice for both diagnosis and treatment. Here, we report one case of PPM with increased fluorodeoxyglucose (FDG) uptake and associated micronodules, which was clinically suspicious for malignancy. The patient was a 60-year-old female who presented with persistent shortness of breath for 1 year. Chest computed tomography showed a 1.5-cm well-circumscribed homogenous nodule in the left upper lobe with increased FDG uptake and multiple smaller well-circumscribed micronodules scattered in both lungs. Left upper lobe wedge resection confirmed the diagnosis of PPM. PPM can deceptively mimic malignancy, so recognizing this rare entity and including it in the differential diagnoses of pulmonary nodules, especially with avid uptake of FDG, is crucial to avoid misdiagnosis and overtreatment.

## INTRODUCTION

Meningioma represents the most common central nervous system (CNS) tumor. Primary extracranial and extraspinal meningiomas are uncommon and account for 2–3% of all meningiomas [1]. Primary pulmonary meningioma (PPM) is a rare entity and under the category of lung tumors of ectopic tissues in World Health Organization (WHO) classification fifth ed. There are <60 cases reported since the first case was described in 1982 [2]. Here, we report a rare case of PPM with an increased fluorodeoxyglucose (FDG) uptake and associated micronodules.

## CASE REPORT

**Figure 1:**
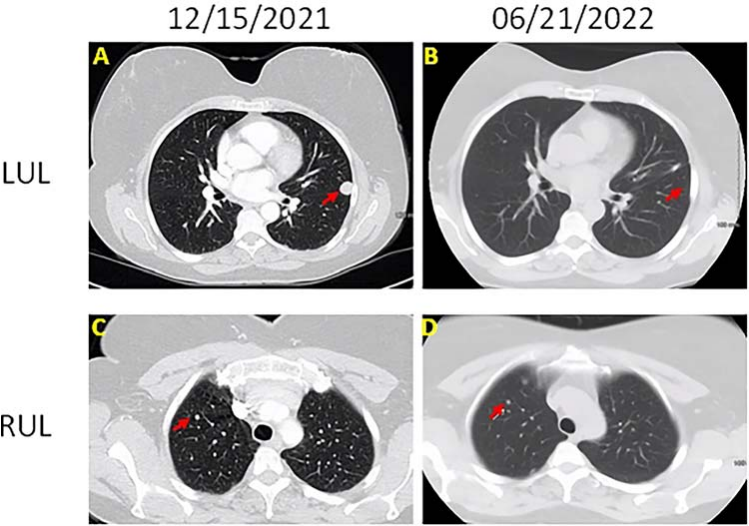
Chest CT images of the lesions (**A**) left upper lobe lesion (1.5 cm) before surgery on 15 December 2021; (**B**) left upper lobe lesion after surgery on 21 June 2022; (**C**) right upper lobe lesion (0.5 cm) on 15 December 2021; (B) right upper lobe lesion on 21 June 2022; there is no change on the size.

The patient was a 60-year-old female who presented with persistent shortness of breath for 1 year. Chest computed tomography (CT; Fig. 1) with contrast showed a 1.5-cm well-circumscribed, homogenous, noncalcified nodule in the left upper lobe and multiple similar, but smaller (3–5 mm), nodules involving right upper, right lower and left lower lobes. Positron emission tomography (PET)-CT revealed that the 1.5-cm nodule was hypermetabolic with SUV of 5.2 and suspicious for malignancy. The other smaller nodules were not detected by PET scan.

**Figure 2:**
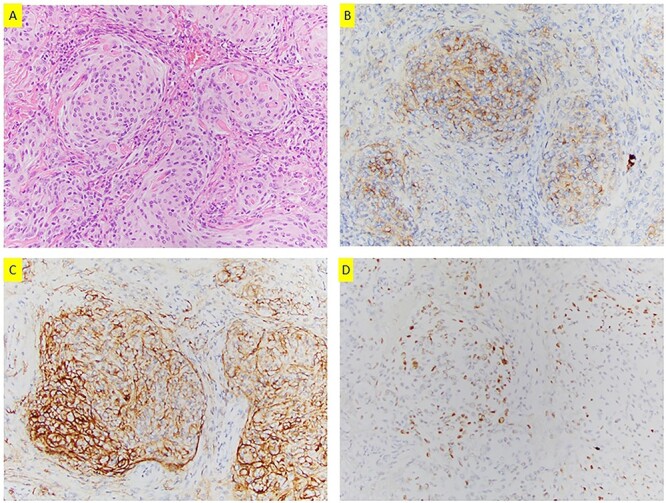
(**A**) H&E section (200×) shows whorls formation and pseudo-nuclear inclusions; tumor cells are positive for EMA (**B**), SSTR2a (**C**) and PR (**D**) (200×).

Left upper lobe wedge resection was performed. On gross examination, the nodule was tan-white, well-circumscribed, firm, homogeneous, measuring 1.5 × 1.3 × 0.7 cm, abutting the pleura. Microscopically, this tumor was composed of bland epithelioid and spindle cells with moderate amount of cytoplasm, whorl formation and occasional pseudo-nuclear inclusions (Fig. 2). No mitoses or necrosis was identified. Tumor cells were positive for epithelial membrane antigen (EMA), progesterone receptor (PR), somatostatin receptor 2a (SSTR2A) (Fig. 2) and S100. CNS meningioma was ruled out clinically. The overall findings support the diagnosis of PPM. The patient was doing well on a 3-month follow-up after wedge resection. All the small nodules remained unchanged on chest CT.

## DISCUSSION

Most cases of PPM are incidental findings on chest X-ray or CT examination. Patients are often asymptomatic or with some nonspecific symptoms [3, 4]. There is a slight female predominance [3]. PPMs are usually 0.6–6 cm in diameter and appear as a solitary, well-circumscribed, homogenous nodule with no anatomic site predilection [5, 6]. Rare cases of multiple nodules, cystic lesions or ground-glass opacities changes have been reported [7–10]. It can have various contrast-enhancement patterns [11] and increased FDG uptake on PET [4, 12–16]. It is impossible to distinguish PPMs from lung metastasis or advanced lung cancer radiologically. Biopsy and cytology were usually not very helpful in the diagnosis of PPMs [4]. The final diagnosis of PPM mainly relies on the pathological examination of resection specimen.

PPMs share the same histomorphology and WHO grading with that of CNS meningioma. It is well circumscribed and consists of epithelioid or spindle cells in whorl pattern. The pseudo-nuclear inclusions or psammoma bodies may present. Tumor cells are positive for EMA, PR and SSTR2A, but negative for TTF, P40, cytokeratin, melanoma markers and neuroendocrine markers [3, 4, 17]. PPMs are most often WHO Grade I with transitional and fibrous patterns. Rare cases with chordoid [15, 18, 19], atypical [14] and rhabdoid features [20] have been reported. Four cases of malignant PPMs have also been documented in literature [21–24].

There are three types of pleuropulmonary meningothelial proliferation, which include metastatic pulmonary meningiomas (MPMs), PPMs and minute pulmonary meningothelial-like nodules (MPMNs). They share similar morphological and immunohistochemical features. Although MPMs is rare, lung is one of the most frequently metastatic sites [25]. Exclusion of MPMs is required for the diagnosis of PPMs. MPMNs are meningothelial-like proliferation in the lung and are present as small solitary or multiple nodules, ranging in size from 1 to 3 mm [26]. They can be distinguished from PPM by their smaller size, frequently perivenular pattern of growth and ill-defined borders [25, 26]. It has been hypothesized that PPMs may originate from MPMNs based on similar morphological, immunohistochemical and ultrastructural features [27–29]. Coexistence of PPM with MPMNs have been reported, favoring this hypothesis [28, 29]. Other hypotheses of PPM etiology have also been proposed such as deriving from ectopic intrapulmonary arachnoid cells [2, 30, 31] or from the pluripotential subpleural mesenchyme [2, 30].

Most PPMs are benign, slow-growing tumors with good prognosis [32]. Wedge resection under thoracoscopy is the first-line treatment and diagnosis. No recurrence or metastasis have been reported in benign PPMs. Conservative treatment was reported in one case with a 2.5-cm biopsy-proven PPM, and the nodule kept unchanged on 7-month follow-up [33]. Malignant PPMs behave very aggressively with recurrence and metastasis even after lobectomy and adjuvant treatment [21–24].

Although tissue diagnosis of PPM does not seem to be challenging based on morphology and immunostaining profile, misdiagnosis and overtreatment do occur. One major reason is unawareness of this rare entity. Misdiagnosis for metastatic papillary thyroid carcinoma can occur in cytology when PPMs present with pseudo-nuclear inclusions and psammoma bodies [17, 29]. PPMs with deceptive increased FDG uptake were reported to be mistaken as lung metastasis in patients with a history of colon cancer and breast cancer [24, 30, 34, 35]. Lobectomy is mostly unnecessary for the treatment of PPMs. However, overtreatment with lobectomy was reported in 11 out of 16 cases of benign PPMs [4]. To avoid misdiagnosis and overtreatment, intraoperative diagnosis is crucial, so it is important to include PPM in the differential diagnoses of indeterminate pulmonary nodules, even the one with avid FDG uptake.

Of note, PPM in our case also presents with associated multiple micronodules, ranging in size from 3 to 5 mm. This manifestation is rare, and very few similar cases have been reported [7–9, 33]. In two of these cases, different nodules were sampled, and histology confirmed the diagnosis of multiple PPM [7, 8]. For other cases, the diagnosis was based on the histology of the largest lesion, and small nodules were on active surveillance [9, 33]. This multiple-nodule presentation can easily be confused with lung metastasis or advanced lung cancer [9].

## CONCLUSION

PPM is a rare entity. Its diagnosis relies on pathology and exclusion of CNS meningioma. Wedge resection is so far the best choice for both diagnosis and treatment. Including PPM in the differential diagnoses of pulmonary nodules, especially the one with increased FDG uptake, can help to avoid misdiagnosis and overtreatment.
